# Mitochondrial DNA copy number in cervical exfoliated cells and risk of cervical cancer among HPV-positive women

**DOI:** 10.1186/s12905-020-01001-w

**Published:** 2020-07-02

**Authors:** Wei Sun, Xueyun Qin, Jing Zhou, Mingjing Xu, Zhangyan Lyu, Xin Li, Kai Zhang, Min Dai, Ni Li, Dong Hang

**Affiliations:** 1 Department of Epidemiology and Biostatistics, School of Public Health, Nanjing Medical University, No. 101 Longmian Ave, Jiangning District, Nanjing, 211166 China; 2 Department of Gynecology, The First Affiliated Hospital of Nanjing Medical University, Nanjing, 210036 China; 3 National Office for Cancer Prevention and Control, Cancer Institute and Hospital, Chinese Academy of Medical Sciences, Beijing, 100021 China; 4 Department of Cancer Prevention, Cancer Institute and Hospital, Chinese Academy of Medical Sciences, Beijing, 100021 China; 5 Jiangsu Key Lab of Cancer Biomarkers, Prevention and Treatment, Collaborative Innovation Center for Cancer Personalized Medicine, Nanjing Medical University, Nanjing, 211166 China

**Keywords:** Mitochondrial DNA copy number, Mitochondrion, Cervical cancer, Human papillomavirus, Case-control study

## Abstract

**Background:**

Although human papillomavirus (HPV) infection has been regarded as the cause of cervical cancer in over 99% of cases, only a small fraction of HPV-infected women develop this malignancy. Emerging evidence suggests that alterations of mitochondrial DNA copy number (mtCN) may contribute to carcinogenesis. However, the relationship between mtCN and cervical cancer remains undetermined.

**Methods:**

The current study included 591 cervical cancer cases and 373 cancer-free controls, all of whom were infected with high-risk HPV. Relative mtCN in cervical cancer exfoliated cells was measured by qRT-PCR assays, and logistic regression analysis was performed to compute odds ratios (ORs) and 95% confidence intervals (CIs). Interaction between mtCN and HPV types was assessed by using the Wald test in logistic regression models.

**Results:**

HPV16, 18, 52, and 58 were the most common types in both case and control groups. Median mtCN in cases was significantly higher than that in controls (1.63 vs. 1.23, *P* = 0.03). After adjustment for age and HPV types, the highest quartile of mtCN was associated with increased odds of having cervical cancer (OR = 1.77, 95% CI = 1.19, 2.62; *P* < 0.01), as compared to the lowest quartile. A dose-response effect of mtCN on cervical cancer was also observed (*P*_trend_ < 0.001). The interaction between mtCN and HPV types was statistically nonsignificant.

**Conclusions:**

In women who test HPV positive, the increase of mtCN in cervical exfoliated cells is associated with cervical cancer. This suggests a potential role of mtCN in cervical carcinogenesis.

## Background

Cervical cancer represents the fourth most common malignancy in women worldwide [1]. Persistent infection with a subset of human papillomavirus (HPV), termed high-risk types, has been recognized as the crucial cause of the disease [2]. As a primary prevention strategy, HPV vaccination against infection is safe and effective to prevent cervical intraepithelial neoplasia (CIN) and cervical cancer [3]. For women who are already infected with HPV, secondary prevention efforts including cervical cancer screening and early intervention can mitigate the incidence and mortality of cervical cancer [4]. Current guidelines for cervical screening are largely based on HPV testing and cytologic diagnosis [5]. However, because HPV infection is a very common event and approximately 90% of infections resolve spontaneously within 2 years, HPV testing is limited by a low positive predictive value (less than 50%) for high-grade lesions [6]. Although cytology-based triage is recommended in cervical screening, this technique has a relatively low sensitivity and high dependence on an experienced cytologist [7]. Therefore, an investigation of new triage approaches for HPV-positive women is important to improve the identification of individuals at high risk of cervical cancer.

Mitochondrial DNA (mtDNA) is an extra-chromosomal circular, double-stranded DNA in eukaryotic cells [8]. It consists of 16.5 kilobase pairs that encode 37 genes involved in various cellular activities, including energy metabolism, free oxygen radical generation, and cell apoptosis [9]. Due to the lack of protective histones, introns, and efficient DNA repair mechanism, mtDNA is particularly vulnerable to reactive oxidative species (ROS) and other sources of genotoxic stress, which may induce mtDNA damage and copy number alterations [10]. Altered mtDNA copy number (mtCN) could affect the expression and function of mitochondrial genes, leading to abnormal cellular metabolism and proliferation [11]. Increasing evidence suggests that mtCN alterations play an important role in pathogenesis of different types of cancer [12]. Compared to adjacent normal tissues, mtCN was significantly increased in head and neck cancer [13], esophageal carcinoma [14], and endometrial carcinoma tissues [15], whereas it was decreased in advanced lung cancer [16], hepatocellular carcinoma [17], gastric cancer [18], and colorectal cancer tissues [19]. Increased mtCN has been considered as a compensation for metabolic defects in impaired mitochondria [20]. When cancer progresses to advanced stages, cumulative damages to mitochondria may elicit mtDNA degradation and decompensation, resulting in a decrease in mtCN and the Warburg effect [21, 22].

High-risk HPV oncoproteins E6 and E7 can induce chronic ROS responses, which are able to promote DNA damage and malignant phenotypes of HPV-infected cells [23, 24]. Increased ROS may also give rise to mtDNA damage and alter mitochondrial abundance in cervical cells. Therefore, we hypothesized that mtCN alterations are implicated in the development of cervical cancer. To our knowledge, no prior studies have assessed the association between mtCN and cervical cancer among HPV-positive women.

In this case-control study, we detected mtCN in cervical exfoliated cells collected from participants who were all high-risk HPV positive. We aimed to provide precursory evidence on the role of mtCN in cervical cancer and its potential as a biomarker for cervical cancer.

## Methods

### Study participants

This study was approved by the ethics committees of Nanjing Medical University and Cancer Institute and Hospital, Chinese Academy of Medical Sciences. All participants provided the written informed consent and thereafter underwent cytological examination. Cervical exfoliated cell specimens were collected by experienced gynecologists using cytobrushes. For those with abnormal cytology, we recommended a colposcopy, during which cervical biopsies were taken. The included cervical cancer patients were histologically confirmed and consecutively recruited from Cancer Institute and Hospital between January 2010 and July 2013, as previously described [25]. Exclusion criteria included those who had recurrent cervical cancer or a history of other malignancies, and those who had received chemo-radio therapy before specimen collection. Of the 5066 women who asked for outpatient gynecological consultation in Cancer Institute and Hospital during the same period and were free of liquid-based cytological abnormalities (atypical squamous cells of undetermined significance, ASCUS, or worse) but were high-risk HPV positive were included as cancer-free individuals. Approximately 12,100 women who attended an outpatients appointment and had an abnormal cervical cytology were excluded. For those participants with normal cytology, a colposcopy was not routinely used unless they were HPV16 or 18-DNA positive. High-risk HPV prevalence was 97.7% (593/607) in cervical cancer patients and 8.0% (407/5066) in cancer-free individuals [25]. In the current study, all HPV16/18-positive controls accepted colposcopy and showed negative results. Due to insufficient DNA, specimens from two patients and 34 cancer-free individuals were excluded. Finally, a total of 591 cancer cases and 373 controls (all high-risk HPV positive) were included in the analysis.

### HPV genotyping

The procedure for HPV genotyping has been described in detail elsewhere [26]. Briefly, total genomic DNA was isolated from cervical exfoliated cells using the QIAamp DNA Mini Kit (Qiagen, Valencia, CA, USA). To evaluate the quality of DNA samples, the β-actin gene was amplified by PCR. Only qualified samples were further tested for HPV DNA by using the HPV GenoArray Test Kit (HybriBio Ltd., Beijing, China). The method could detect a total of 21 HPV types simultaneously, including 13 high-risk types (HPV16, 18, 31, 33, 35, 39, 45, 51, 52, 56, 58, 59, and 68), two intermediate-risk types (HPV53 and 66), and six low-risk HPV types (HPV6, 11, 42, 43, 44, and 81).

### Measurement of mtDNA copy numbe*r*

Relative mtCN in cervical exfoliated cells was determined by real-time quantitative PCR (qRT-PCR), as described in our previous study [27]. In brief, we used two pairs of specific primers that were designed to amplify the partial regions of mitochondrial subunit ND1 gene (*MT-ND1*, Forward: 5′-CCCTAAAACCCGCCACATCT-3′; Reverse: 5′-GAGCGATGGTGAGAGCTAAGGT-3′) and the single-copy nuclear gene human globulin (*HGB*, Forward: 5′-GAAGAGCCAAGGACAGGTAC-3′; Reverse: 5′-CAACTTCATCCACGTTCACC-3′), respectively. The qRT-PCR reaction was performed using the SYBR Green Realtime PCR Master Mix (Toyobo Co. Ltd., Osaka, Japan) on the 7900HT Real-Time PCR System (Applied Biosystems, CA). All assays were done in triplicates in 384-well plates, and laboratory technicians were blinded to the case-control status during the experiments. To assess the inter-plate variation in PCR efficiency, for each 384-well plate, DNA samples from five randomly selected controls were equally pooled as the reference and serially diluted 1:2 (20 to 0.625 ng/μl) to produce a standard curve, which showed a high coefficient of determination (above 0.99) for each reaction. We calculated the ratio of *MT-ND1* to *HGB* (−dCt) for each sample by subtracting the average *HGB* Ct value from the average *MT-ND1* Ct value. The relative ratio of *MT-ND1* to *HGB* (−ΔΔCt) was computed by subtracting the ratio of *MT-ND1* to *HGB* of the calibrator DNA from the ratio of each sample. Relative mtCN was calculated by using the formula 2^-ΔΔCt^ [27]. The average inter- and intra-plate variations, determined by the measurement of quality control samples, were 1.9 and 1.0%, respectively.

### Statistical analysis

Pearson χ^2^ tests were used to evaluate the differences in demographic characteristics and HPV types between cases and controls. Unconditional logistic regression was performed to estimate the odds ratio (OR) and 95% confidence intervals (CIs) for cervical cancer in each mtCN quartile, with the adjustment for age and HPV types. Statistical significance of interaction was assessed by a Wald test for the cross-product terms between mtCN and HPV types in logistic regression models. A two-tailed *P* value of less than 0.05 was considered statistically significant. All statistical analyses were conducted by using R (version 3.3.3).

## Results

**Fig. 1:**
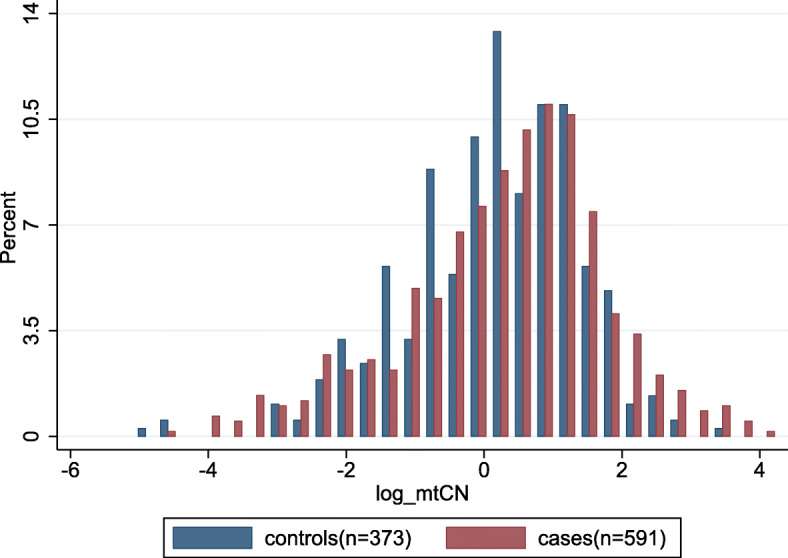
Distribution of mitochondrial copy number (log-transformed) in the cases and controls

Table 1 shows the demographic and clinical characteristics of participants. In both cases and controls, HPV16, 18, 52, and 58 were the most frequent types. No statistically significant differences were observed in age, HPV18, and multiple infections between the case and control groups. However, cervical cancer patients had a higher proportion of HPV16 infection (54.65% vs. 19.57%, *P* < 0.001) but lower proportions of HPV52 (17.43% vs. 27.08%, *P* < 0.001) and HPV58 (9.31% vs. 15.55%, *P* = 0.005), as compared to the control group. More importantly, the median mtCN was higher in cervical cancer cases than that in the controls (1.63 vs. 1.23, *P* = 0.03). Most of the cases were squamous carcinoma (94.9%) and at FIGO I/II stage (83.8%). Figure 1 shows the distribution of mtCN in the cases and controls.

**Table 1:** Basic characteristics of cervical cancer cases and cancer-free controls

Variables	Cases (*n* = 591)	Controls (*n* = 373)	*P*
	N (%)	N (%)	
Age, years			0.36 ^**e**^
< 50	339(57.36)	226(60.59)	
≥ 50	252(42.64)	147(39.41)	
HPV16			**< 0.001**^**e**^
Negative	268(45.35)	300(80.43)	
Positive	323(54.65)	73(19.57)	
HPV18			0.43 ^**e**^
Negative	532(90.02)	342(91.69)	
Positive	59(9.98)	31(8.31)	
HPV52			**< 0.001**^**e**^
Negative	488(82.57)	272(72.92)	
Positive	103(17.43)	101(27.08)	
HPV58			**0.005**^**e**^
Negative	536(90.69)	315(84.45)	
Positive	55(9.31)	58(15.55)	
Other type ^a^			**< 0.001**^**e**^
Negative	481(81.39)	239(64.08)	
Positive	110(18.61)	134(35.92)	
Multiple infection			0.15^**e**^
Negative	538(91.03)	350(93.83)	
Positive	53(8.97)	23(6.17)	
Grade ^b^			
High	27(7.99)		
Middle	178(52.66)		
Low	133(39.35)		
Histology ^c^			
Squamous	536(94.87)		
Adeno/adenosquamous	29(5.13)		
FIGO ^d^			
I/II	366(83.75)		
III/IV	71(16.25)		
mtCN (median, quartile)	1.63(0.59–3.43)	1.23(0.53–2.76)	**0.03**^f^

Bold values indicate *P* < 0.05

*Abbreviation*: *HPV* human papillomavirus, *FIGO* International Federation of Gynecology and Obstetrics, *mtCN* mitochondrial DNA copy number

^a^Others include HPV31, 33, 35, 39, 45, 51, 56, 59, 66, and 68

^b^Grade information was available in 338 cervical cancer cases

^c^Histological information was available in 565 cases

^d^FIGO information was available in 437 cervical cancer cases

^e^Derived from χ^2^ test

^f^Derived from Wilcoxon rank-sum test

In Table 2, compared with the lowest quartile of mtCN, a significantly increased odds of having cervical cancer was observed in the third (OR = 1.56, 95% CI: 1.04, 2.33; *P* = 0.03) and fourth quartile groups (OR = 1.77, 95% CI: 1.19, 2.62; *P* = 0.004). A dose-response effect of mtCN on cervical cancer was statistically significant (*P*_trend_ < 0.001).

**Table 2:** Association between mitochondrial DNA copy number and cervical cancer

mtCN	Cases (%)	Controls (%)	OR (95% CI) ^a^	*P*^a^	*P*_trend_
≤0.53	135(22.84)	94(25.21)	1.00		**< 0.001**
0.53–1.23	110(18.61)	93(24.93)	1.03(0.67,1.56)	0.90	
1.23–2.76	154(26.06)	92(24.66)	1.56(1.04,2.33)	**0.03**	
≥2.76	192(32.49)	94(25.20)	1.77(1.19,2.62)	**0.004**	

Bold values indicate *P* < 0.05

*Abbreviation*: *mtCN* mitochondrial DNA copy number, *OR* odds ratio, *CI* confidential interval

^a^Derived from logistic regression with adjustment for age, HPV 16, 18, 52,58 and the other high-risk type infection status

We performed the stratified analysis by age and different HPV types (Table 3). The dose-response effect of mtCN on cervical cancer risk was largely consistent between subgroups, with the interaction between mtCN and the stratified variables statistically nonsignificant (all *P* > 0.05).

**Table 3:** Association between mitochondrial DNA copy number and cervical cancer

Variables	Quartiles of mtCN ^a^				*P*_trend_	*P*_interaction_^c^
	1st quartile	2nd quartile	3rd quartile	4th quartile		
	Reference	OR (95% CI) ^b^	OR (95% CI) ^b^	OR (95% CI) ^b^		
Age (years)						
< 50	1.00	0.87(0.49–1.55)	1.25(0.74–2.10)	1.44(0.87–2.40)	0.08	0.40
≥ 50	1.00	1.14(0.61–2.16)	2.10(1.10–4.02)	2.32(1.24–4.35)	**0.002**	
HPV16						
Negative	1.00	1.10(0.67–1.89)	2.09(1.26–3.46)	2.03(1.23–3.33)	**0.001**	0.21
Positive	1.00	1.00(0.55–2.10)	0.79(0.40–1.57)	1.56(0.77–3.16)	0.34	
HPV18						
Negative	1.00	1.08(0.74–1.68)	1.57(1.02–2.41)	1.67(1.10–2.53)	**0.01**	0.26
Positive	1.00	0.56(0.26–2.47)	1.30(0.38–4.50)	2.97(0.77–11.38)	0.07	
HPV52						
Negative	1.00	0.97(0.71–1.55)	1.46(0.93–2.3)	1.88(1.21–2.92)	**0.002**	0.77
Positive	1.00	1.31(0.44–3.43)	1.85(0.71–4.8)	1.67(0.65–4.28)	0.24	
HPV58						
Negative	1.00	1.04(0.73–1.62)	1.54(1.00–2.37)	1.71(1.12–2.61)	**0.003**	0.65
Positive	1.00	0.58(0.27–2.44)	1.65(0.50–5.49)	2.15(0.69–6.67)	0.08	
Others						
Negative	1.00	1.22(0.66–2.12)	1.14(0.68–1.90)	1.80(1.10–2.96)	**0.03**	0.25
Positive	1.00	0.81(0.57–1.63)	2.40(1.22–4.71)	1.94(0.99–3.81)	**0.003**	
Multiple infection						
Negative	1.00	1.10(0.74–1.71)	1.57(1.03–2.39)	1.82(1.21–2.74)	**0.001**	0.25
Positive	1.00	0.26(0.16–1.82)	1.07(0.18–6.37)	1.11(0.19–6.56)	0.42	

Bold values indicate *P* < 0.05

*Abbreviation*: *HPV* human papillomavirus, *mtCN* mitochondrial DNA copy number, *OR* odds ratio, *CI* confidential interval

^a^Quartiles of mtDNA copy number were assigned based on the distribution among controls

^b^Derived from logistic regression with adjustment for age, HPV 16, 18, 52, 58 and the other high-risk HPV types where appropriate

^c^Interaction analysis was conducted by adding a multiplicative interaction term in unconditional logistic regression models

## Discussion

In this study, relative mtCN was determined in cervical exfoliated cells from 591 cervical cancer patients and 373 cancer-free controls. We found that median mtCN in the patients was significantly higher than that in the controls. After adjustment for age and HPV types, a higher level of mtCN remained associated with increased odds of having cervical cancer, and there was a dose-response effect of mtCN on cervical cancer. The results suggest that mtCN alterations may be implicated in cervical carcinogenesis and might represent a potential biomarker for triage of HPV-positive women.

To date, few studies have investigated the association between mtDNA abundance and cervical cancer. Based on tissue samples, Warowicka et al. showed that total mtCN was cumulatively increased in high-grade squamous intraepithelial lesion (*n* = 30) and cervical cancer (*n* = 29), compared to that in low-grade lesion samples (*n* = 29) [28], whereas Kabekkodu et al. reported a lower level of mtCN in cervical cancer tissues (*n* = 20) than in cervicitis samples (*n* = 10) [29]. Small sample sizes and different cancer stages might partly explain the inconsistency. Moreover, neither of the studies detected HPV infection, which is a pre-requisite for cervical cancer. In fact, cervical exfoliated cells are collected in cytology-based cervical screening and may also be a source of molecular biomarkers indicative of neoplastic changes in the underlying tissue. In the current study, for the first time we determined HPV types and mtCN in cervical exfoliated cells from cervical cancer patients and healthy controls. We found that mtCN in cervical cells was positively associated with cervical cancer after adjustment for age and HPV types. However, given the relatively weak association with cervical cancer, a combination with other biomarkers, such as E6/E7 mRNA, p16INK4a-Ki-67, and HPV integration [30, 31], may improve the prediction of cervical cancer risk for HPV-positive women. In addition, it has been suggested that higher mtCN in cervical cancer tissues is associated with a reduced overall survival, implying that alterations in mtDNA content might also affect cervical cancer progression [32].

Furthermore, many epidemiological studies have explored the association of mtCN in peripheral blood leukocytes with different types of cancer, leading to quite conflicting results [33]. A meta-analysis including 36 case-control studies showed a positive association of blood mtCN with lymphoma and breast cancer, and a negative association for hepatic carcinoma [34]. It remains unclear whether the variation of mtCN in blood can really reflect the etiology of a specific cancer.

High-risk HPV infection may cause a series of mitochondrial dysfunction by accelerating the production of ROS [35, 36]. Warowicka et al. observed that both mtCN and ROS were increased during cervical cancer development [28]. Although HPV infection usually does not mount an inflammatory response, viral oncogenes can induce a chronic ROS response. In vitro studies showed that the expression of E6*, a truncated isoform of HPV16 E6 protein, increased ROS levels in cervical cancer cells [23]. HPV16 E6 and E7 proteins can also evoke a ROS response via NOX2 oxidase activation [24]. Additionally, HPV18 E2 protein has been demonstrated to localize to mitochondrial membranes and augment mitochondrial ROS production without cell death, whereas low-risk HPV6 E2 exhibits very low interaction to mitochondria [37]. Increased ROS are thought to cause mtDNA injuries and initiate mtDNA replication to counterbalance functional defects in impaired mitochondria [38, 39]. Another possibility is that specific genetic events in the D-loop region, i.e., the non-coding mtDNA region which contains crucial elements for replication, may lead to the up-regulation of mtDNA replication [15]. Due to major roles of normal mitochondria in energy production, metabolism, and apoptosis, the accumulation of mtDNA alterations may contribute to the pathogenesis of cervical cancer.

Furthermore, several studies point out that tumor suppressor p53 can regulate mtCN and mitochondrial biogenesis [40, 41], inhibit the mitochondrial damage induced by ROS [42], and participate in the regulation of mitochondrial respiration [43]. High-risk HPV E6 protein has the ability to induce p53 degradation via the ubiquitin-proteasome pathway [44, 45].. Consequently, high-risk HPV E6 may disorder the p53 functions and thereby cause mtCN alterations. The interplay of HPV, p53, ROS, and mitochondria warrants further investigations to uncover the mechanism underlying cervical cancer.

Although this study suggests a potential role of mtCN in cervical carcinogenesis, several limitations should be addressed. First, due to the retrospective nature of a case-control study, a causal relationship remained to be established. Second, among the controls, only those with HPV16/18 positivity underwent colposcopy and showed normal findings. A possibility of cervical precursor lesions in the remaining controls cannot be completely excluded. Finally, although the interaction between mtCN and HPV types was statistically nonsignificant, our interaction analysis was possibly underpowered.

## Conclusions

Our study indicates a positive association between mtCN and cervical cancer, suggesting that mtCN alteration may play a role in cervical carcinogenesis. Prospective studies are needed to establish the causal relationship and determine when the association becomes detectable in the pathogenetic process.

## Data Availability

All datasets analyzed in this study are available from the corresponding author on reasonable request.

## Abbreviations

HPV: Human papillomavirus
mtCN: Mitochondrial DNA copy number
OR: Odds ratio
CI: Confidence interval
CIN: Cervical intraepithelial neoplasia
mtDNA: mitochondrial DNA
ROS: Reactive oxidative species
ASCUS: Atypical squamous cells of undetermined significance
qRT-PCR: real-time quantitative PCR
MT-ND1: Mitochondrial subunit ND1 gene
HGB: Human globulin
FIGO: International Federation of Gynecology and Obstetrics
